# Molecular cloning, structure, phylogeny and expression analysis of the invertase gene family in sugarcane

**DOI:** 10.1186/s12870-017-1052-0

**Published:** 2017-06-23

**Authors:** Liming Wang, Yuexia Zheng, Shihui Ding, Qing Zhang, Youqiang Chen, Jisen Zhang

**Affiliations:** 1 Center for Genomics and Biotechnology, Fujian Provincial Key Laboratory of Haixia Applied Plant Systems Biology, Haixia Institute of Science and Technology (HIST), Fujian Agriculture and Forestry University, Fuzhou, 350002 China; 2 College of Life Science, Fujian Agriculture and Forestry University, Fuzhou, 35002 China; 3 College of Life Sciences, Fujian Normal University, Fuzhou, 350117 China; 4 Key Laboratory of Sugarcane Biology and Genetic Breeding Ministry of Agriculture, Fujian Agriculture and Forestry University, Fuzhou, 350002 China

**Keywords:** Sugarcane, Invertase, Gene expression pattern, Abiotic stress, Quantitative RT-PCR

## Abstract

**Background:**

Invertases (INVs) are key enzymes regulating sucrose metabolism and are here revealed to be involved in responses to environmental stress in plants. To date, individual members of the invertase gene family and their expression patterns are unknown in sugarcane due to its complex genome despite their significance in sucrose metabolism.

**Results:**

In this study, based on comparative genomics, eleven cDNA and twelve DNA sequences belonging to 14 non-redundant members of the invertase gene family were successfully cloned from sugarcane. A comprehensive analysis of the invertase gene family was carried out, including gene structures, phylogenetic relationships, functional domains, conserved motifs of proteins. The results revealed that the 14 invertase members from sugarcane could be clustered into three subfamilies, including 6 neutral/alkaline invertases (ShN/AINVs), and 8 acid invertases (ShAINVs). Faster divergence occurred in acid INVs than in neutral/alkaline INVs after the split of sugarcane and sorghum. At least a one-time gene duplication event was observed to have occurred in the four groups of acid INVs, whereas ShN/AINV1 and ShN/AINV2 in the β8 lineage were revealed to be the most recently duplicated genes among their paralogous genes in the β group of N/AINVs. Furthermore, comprehensive expression analysis of these genes was performed in sugarcane seedlings subjected to five abiotic stresses (drought, low temperature, glucose, fructose, and sucrose) using Quantitative Real-time PCR. The results suggested a functional divergence of INVs and their potential role in response to the five different treatments. Enzymatic activity in sugarcane seedlings was detected under five abiotic stresses treatments, and showed that the activities of all INVs were significantly inhibited in response to five different abiotic stresses, and that the neutral/alkaline INVs played a more prominent role in abiotic stresses than the acid INVs.

**Conclusions:**

In this study, we determined the INV gene family members of sugarcane by PCR cloning using sorghum as a reference, providing the first study of the INV gene family in sugarcane. Combining existing INV gene data from 7 plants with a comparative approach including a series of comprehensive analyses to isolate and identify INV gene family members proved to be highly successful. Moreover, the expression levels of INV genes and the variation of enzymatic activities associated with drought, low temperature, glucose, fructose, and sucrose are reported in sugarcane for the first time. The results offered useful foundation and framework for future research for understanding the physiological roles of INVs for sucrose accumulation in sugarcane.

**Electronic supplementary material:**

The online version of this article (doi:10.1186/s12870-017-1052-0) contains supplementary material, which is available to authorized users.

## Background

Sugarcane is one of the most economically valuable crops worldwide and accounts for up to 80% of the global sucrose production. It serves as an important model crop to study sucrose accumulation due to its remarkable ability to accumulate large amounts of sucrose in its stems that can reach close to 700 mM or in excess of 50% of the dry weight (DW) [33]. With the increasing demand due to biofuel production and challenges with biomass production [7], more and more attention has been devoted to increase sucrose yield in sugarcane. Therefore, the mechanism of sucrose accumulation in sugarcane is now considered to be one of the top priorities in sugarcane research. However, the modern sugarcane cultivar has one of the most complex genomes being both aneupoid and autopolypoid with an extreme ploidy level ranging from octoploid (x = 8) to dodecaploid (x = 12). To date, the studies of the genes in sucrose metabolism have been especially limited.

In plants, invertases (EC 3.2.1.26, INV) catalyze the irreversible hydrolysis of sucrose into glucose and fructose, and are thus considered to be a pivotal enzyme in the regulation of sucrose metabolism [4]. In addition, INVs have also been demonstrated to contribute to numerous aspects of plant growth and development [49, 51], organ formation, sugar transport, stress response [56], carbon partitioning [41, 51], phloem unloading and source/sink regulation [37], and adjusting the composition and levels of sugar in sink tissue [37]. Plant INVs are encoded by large genes families, which can be divided into an acid INV sub-family and a neutral/alkaline INV sub-family according to their optimal pH for activity [46, 55]. The acid INV subfamily includes cell wall invertases (CWINVs) as cell wall-bound forms and vacuole invertases (VINVs) as soluble forms [4]; cell wall invertase originated from respiratory eukaryotes, whereas vacuole invertases derive from aerobic bacteria [47]. Vacuole invertases as soluble acid invertases (SAINVs) generally branch off from cell wall-bound acid invertases in evolution [23]. Cell wall INVs may play versatile regulatory roles in reproductive development, phloem unloading, carbon partitioning [41, 51] and sink development [10, 46]. Vacuolar INVs regulate osmotic pressure, sugar signals, sucrose accumulation, and sucrose concentration especially during the expansion phases of sink organs [25]. The gene structure of acid invertases (AINVs) is highly conserved and contains six to eight exons. In almost all acid invertase genes, the second exon codes for only three amino acids, DPN, belonging to the conserved NDPNG motif of the catalytic domain, and is the smallest functional motif known in plant biology [1]. The molecular weights of mature acid invertases, which are N-glycosylated at multiple sites, range from 55 to 70 kD [52]. Acid invertases contain an N-terminal domain structure, a mature polypeptide and a C-terminal region [46]. The N-terminal domain structure comprises a signal peptide and a propeptide that is in total about 100 amino acid residues long [47]. Neutral/alkaline invertases (N/AINVs), which only exist in plants and in photosynthetic bacteria, are believed to have originated from cyanobacteria [4]. In contrast to acid INVs, neutral/alkaline INVs localize to multiple subcellular compartments including mitochondria, plastids [34], and the nucleus [41]. Neutral/alkaline INVs differ from acid INVs as they do not contain an N-terminal signal peptide, are not glycosylated [36], and are therefore less stable [39]. The role of neutral/alkaline INVs is less clear than that of acid INVs [42]. So far, extensive characterization of invertases including cDNAs, protein purification and/or genes have been reported from several plants including agave, rice, wheat, tomato, carrot, maize, *Populus trichocarpa* and Arabidopsis [4, 9–11, 16, 23, 27, 30, 46, 47, 53].

In sugarcane, the enzyme invertases are well documented key regulators of the accumulation of sucrose in the stems [13, 17, 18, 58]; N/AINVs are found in the cytoplasm or in metabolic compartments of cells, have low activity in meristematic cells, and are involved in sugar accumulation in storage tissue [17]. In 18 month-old sugarcane cultivars, N/AINVs have been suggested to be more important in sucrose hydrolysis than SAINVs in the early storage of sugar in the stems after harvest [44]. Recently, a reduction of N/AINVs activity in transgenic sugarcane plants was revealed to cause a decrease in respiration and sucrose cycling, and an increase in the sucrose to hexose ratio, demonstrating the essentiality of N/AINVs in directing carbon towards respiratory processes in the sugarcane culm [40]. SAINVs were found primarily in the vacuoles of storage parenchyma cells [13, 18], and presented high activity in tissues that are rapidly growing [17]. Sucrose accumulation in the sugarcane stalk has been suggested to be regulated by the difference between the activities of SAINVs and sucrose phosphate synthase [58]. A positive correlation between the changes in transcript levels and enzyme activity in sugarcane cultivars, and between SAINV activity and content of hexose sugars was observed, whereas a negative correlation was found between SAINV activity and sucrose content in mature and immature internodes [44]. In transgenic sugarcane, the intracellular and extracellular sugar composition was highly sensitive to the changes in INV activity, and acid INV activity was negatively correlated to sucrose accumulation [14]; in another study of transgenic sugarcane, 70% reduction in the level of acid INV activity did not alter sucrose load or purity [5].

Although these long-term studies implicated INVs as the principal enzymes regulating sugarcane growth and sucrose accumulation, elucidating the molecular mechanism for invertase function lags behind the guidelines of sucrose genetic modification in sugarcane. A comprehensive understanding of the molecular mechanism and evolution of the gene family in a plant species is the first key step to understand the physiological roles and metabolic mechanism regulated by invertases. The available genome of *Sorghum bicolor*, the closest diploid relative of sugarcane in the Andropogonae tribe, provides an excellent model for sugarcane genomic studies [35]. In this study, the invertase gene members were predicted, based on comparative genomics approaches, further verified by PCR cloning and sequencing, and gene expression levels were investigated by real time PCR. The aims of this study were to: (1) identify the members of the invertase gene family in sugarcane; (2) characterize the expression patterns of the invertase gene family under different abiotic stresses and (3) analyze the genetic diversity and the function to differentiate invertase gene families.

## Methods

### Plant material

Sugarcane cultivar FN41 was maintained in the campus of Fujian Agriculture and Forestry University (Fuzhou, China). According to the method of Moore [32], fresh leaf tissue (the third mature leaf, counted from the leaf rolls) was harvested from 7 to 9-month-old field-grown sugarcane plants for DNA and RNA isolation, which were further used for genomic and cDNA cloning of INV genes

Sugarcane seedlings from callus culture at four leaf stage were used for PEG treatment, cold treatment and sugar treatment experiments. To avoid the background effect of stalk storage nutrition, the seedling plants were recovered using sugar-free MS culture solution for 24 h prior to experimental treatment. PEG stress treatment: seedlings were incubated in sugar-free culture solution containing 10% (*W*/*V*) PEG6000 for a photoperiod of 16-h light at 28 °C/ 8-h dark at 24 °C. Low temperature treatment: seedlings were incubated in sugar-free culture solution and grown in 16 h light at 15 °C/8 h dark at 10 °C. Sugar treatment: three groups of seedlings were incubated in MS culture solution for 4 h dark at 24 °C with 3% (*W*/W) sucrose, 3% (*W*/W) glucose, 3% (*W*/W) fructose respectively. Control treatment: seedlings were incubated in sugar-free culture solution for a photoperiod of 16 h light at 28 °C/8 h dark at 24 °C. Fresh leaf tissue from each of the five treatment experiments was immediately snap-frozen in liquid nitrogen and stored at −80 °C prior to RNA isolation and enzymes extraction. RNA was used for RT-qPCR analysis of INVs’ gene expression.

### BLAST searches of the INV gene families in seven plant species

Genomic sequences of seventeen known INV genes (Additional file 1: Table S1) from *Arabidopsis* (http://www.arabidopsis.org/) and nineteen from rice [23] (Additional file 1: Table S1) were used as queries to search the full set of INV genes in the genomes of grape, papaya, *Brachypodium distachyon*, maize and sorghum. BLAST matches achieved similarity scores of >50.0 and probability scores of <10^−4^ were collected as candidate sequences. These candidate sequences of INV genes were further verified by their annotated database (http://www.phytozome.net/) through BLAST and BLASTX. Furthermore, candidate INV proteins were confirmed by searching for conserved domains of invertase using CD-Search tool (http://www.ncbi.nlm.nih.gov/Structure/cdd/docs/cdd_search.html).

### Cloning of sugarcane invertase genes

To obtain the ORF and DNA sequence of the sugarcane INV genes, primer pairs were designed based on the pile up of the INV gene sequences from sorghum and sugarcane EST resource from Genbank (http://www.ncbi.nlm.nih.gov/genbank/). A forward primer and reverse primer were designed to be at opposite ends of the ORF (Additional file 2: Table S2, available as Supplementary Material to this paper).

Total RNA was extracted using TRIzol reagent (Invitrogen Co., Carlsbad, CA, USA) from fresh leaf samples of mature sugarcane from the field, then treated with RNase-free DNaseI (Ambion, AM1906) prior to being used for reverse transcription. Integrity of the RNA sample was analyzed by agarose gel electrophoresis. Genomic DNA was isolated from fresh leaf samples in mature sugarcane according to the TIANcombi DNA Lyse&Det PCR Kit manufacturer’s instructions (TIANGEN, China).

For cDNA cloning, the first-strand cDNA was synthesized from 1 g of total RNA according to the instructions of the Revert Aid™ First Strand cDNA Synthesis Kit (Thermo Fisher Scientific.). cDNA fragments covering the whole open reading frame of INV genes were amplified by PCR using gene specific primers. Similar PCR protocols were used for both RT-PCR and PCR for genomic DNA. PCR was performed in a 10 μL reaction volume containing 1 μL template of total DNA or first-strand cDNA, 5 μL 2 × GC LA Taq Buffer, 0.2 μL of each PCR primer, 0.2 μL LA Taq, 0.8 μL dNTP (2.5 mmol/L) and 2.6 μL ddH_2_O. The amplified DNA fragments and cDNA fragments were cloned into pMD19-T Vector Kit (TaKaRa) and subsequently sequenced by BGI Tech Solutions Co., Ltd. (BGI-Tech).

### Sequence analysis of invertase family members

Gene annotation: The cloning sequences of potential INVs were BLAST/BLASTx to Genbank database. The determined genomic sequences were annotated with the FGENESH program (http://www.softberry.ru/berry.phtml) with references of cDNA sequences and EST of *Saccharum*. The annotated genes were further manually examined.

Sequencing analysis: The sequences of genomic and cDNA were BLAST/BLASTX to Genbank to confirm that these sequences were INVs. The determined cDNA sequences were translated into protein sequences using the online tool (http://web.expasy.org/translate/). Furthermore, their theoretical isoelectric point (pI) and molecular weight (Mw) were analyzed (http://web.expasy.org/compute_pi/). The putative conserved domains were detected using the CD-Search tool (http://www.ncbi.nlm.nih.gov/Structure/cdd/docs/cdd_search.html), and subcellular localizations were predicted by the subcellular location Prediction Servers (Plant-mPLoc, http://www.csbio.sjtu.edu.cn/bioinf/plant-multi/; SignalP 4.1 Server, http://www.cbs.dtu.dk/services/SignalP/; MitoProt, http://ihg.gsf.de/ihg/mitoprot.html; ChloroP 1.1 Server, http://www.cbs.dtu.dk/services/ChloroP/). The motifs of INV proteins were analyzed by using MEME (http://alternate.meme-suite.org/tools/meme, MEME Suite 4.10.1) with the parameters of maximum motif number with 15, minimum motif width with 6, maximum motif width with 50, and distribution of motif occurrences with Zero or one per sequence. The gene schematic structures were drawn by using the Gene Structure Display Server (http://gsds.cbi.pku.edu.cn/index.php) [19].

### Phylogenetic analysis

The amino acid sequences of INV genes from eight plant species (Additional file 1: Table S1) were used to construct an unrooted phylogenetic tree by MEGA5.1 [50]. Neighbor-joining topologies were generated as the consensus of 1000 bootstrap alignment replicates by running MEGA 5.2 with ClustalW alignment.

### RT-qPCR analysis of gene expressions

1 μg of total RNA from each sample of different treatments was reverse transcribed using the RevertAid™ First Strand cDNA Synthesis Kit (Fermentas). Based on the annotated sugarcane *INV*s genomic sequences, RT-qPCR primers (Additional file 3: Table S3) were designed using software program Beacon Designer 7 to amplify sequences spanning at least one intron, and primers’ specificity was tested via regular RT-PCR for experimental quality control. Real-time PCR was performed in three technical replicates from three biological replicates. To determine the amplification efficiency for each primer set, the calibration curve for each gene was obtained by performing real-time PCR with four dilutions of cDNA (4^0^,4^−1^,4^−2^,4^−3^,4^−4^). RT-qPCR was performed in ABI Prism®7300HT Fast Real-Time PCR machine (Applied Biosystems, USA). PCR reactions contained 10 μL of 2X SYBR Green Master Mix (Takara), 2 μL of template cDNA (10X dilution), 0.4 μL of primers mixed (20 mM of each) and 7.6 μL ddH_2_O. The PCR cycle was: 3 min at 95 °C followed by 40 cycles of 95 °C for 15 s and 60 °C for 45 s and the specificity of the individual PCR amplification was checked using a heat dissociation protocol from 65 to 95 °C following the final cycle of the PCR. 25SrRNA (E1:5′-CCTATTGGTGGGTGAACAATCC-3′; E2:5′-GCAGCCAAGCGTTCATAGC-3′) were used as reference gene, which was verified to exhibit stable levels of expression in a broad range of sugarcane tissues [15, 20, 31]. All the genes from each sample were compared with the expression level of 25SrRNA from leaves of sugarcane and the relative expression level of each INV in different treatments was calculated based on normalized relative quantities.

### Invertase activity

#### Extraction of enzymes

The leaf tissue from each sample of the different treatments was ground in liquid nitrogen and the subsequent procedure for sample extraction was conducted at 4 °C or lower.

#### Extraction of soluble INVs

0.5 g tissues were homogenized with 5 ml 50 mmol/L HEPES extraction buffer, containing of 12 mmol/L MgCl_2_, 1 mmol/L EDTA, 1 mmol/L EGTA,10 mmol/L DTT, 2 mmol/L benzamidine,0.05% Triton-X 100,0.05% BSA,2% PVPP [58]. Homogenates were filtered through a microfiltration membrane and were centrifuged at 9366 g for 10 min. The supernatant was desalted and de-sugared immediately using Sephadex G-25 (Pharmacia PD-10) and kept on ice until the assay was performed.

#### Extraction of cell wall INVs

0.5 g sugarcane leaf tissue was homogenized with 10 ml 50 mmol/L HEPES buffer excluding 2% PVPP) [58] and kept on ice for 10 min. Homogenates were centrifuged at 25151 g rpm for 15 min. The sediments (containing the cell wall fraction) were homogenized with 1.7 ml 50 mmol/L HEPES buffer(the same as above, and were subsequently centrifuged at 25151 g for 15 min at. The supernatants (enriched for the CWI fractions) were desalted twice using Sephadex G-25 (Pharmacia PD-10) and kept on ice until use.

#### INV activity assay

Similarly to Tang et al. [51], 0.4 mL of desalted extracts were homogenized at 37 °C with the reaction mixture (2.4 mL) (1.2 mL 0.1 mol/L phosphate/citric and 0.8 mL 0.1 mol/L sucrose, pH 4.6 for acid INV and pH 7.5 for neutral/alkaline INV) and incubated for 60 min. For the cell wall INV activity assay, 1 mL of desalted extracts were homogenized with 6 mL reaction mixture (3 mL 0.1 mol/L phosphate/citric buffer (pH 4.6), 2 mL 0.1 mol/L of desalted extracts sucrose) and incubated at 37 °C for 120 min. The boiled desalted extracts and concentration gradient of glucose (Sigma-Aldrich) were used as background control and standard, respectively. The reaction was stopped by adding 2.4 mL DNS followed by boiling for 5 min. The liberated reducing sugars were quantified by measuring the absorbance at 540 nm. Micrograms of product formed per gram of total protein per minute(μg.Glc.g-1.Pr.min-1)were used as the enzymatic activity units.

## Results

### Identification of INV genes in the genomes of six plant species

To obtain the reference sequence of the INV gene in sugarcane for comparative genomics analysis, 19 and 17 well-annotated *INV*s from *Oryza sativa* [23] and *Arabidopsis thaliana* (https://www.arabidopsis.org) respectively were used to search these family members from *Vitis vinifera*, *Carica papaya*, *Brachypodium distachyon*, *Zea mays* and *Sorghum bicolor* (Additional file 1: Table S1). 17 *INV*s were found from *Vitis vinifera*, 8 from *Carica papaya*, 19 from *Brachypodium distachyon*, 21 from *Zea mays,* and 19 from *Sorghum bicolor* (Additional file 1: Table S1). The conserved domains and the chromosomal location of these INV genes from seven plant species (including rice and *Arabidopsis*) were analyzed and are listed in Additional file 1: Table S1.

Being the closest diploid relative of sugarcane, sorghum INVs (referred from here on as *SbINV*s) (Additional file 1: Table S1) are described specifically here for further references. In our study, genome-wide identification of the INV gene family in sorghum revealed that there are 19 INVs in the sorghum genome. Of the 19 SbINVs, seven are neutral/alkaline INVs (*SbN/AINV*s), twelve are acid INVs containing ten cell wall INVs (*SbCWINV*s), and two vacuolar INVs (*SbVINV*s). The seven SbN/AINV proteins contain a conserved domain of Glyco_hydro_100, while acid INV proteins contain a conserved domain of both Glyco_hydro_32 N and Glyco_hydro_32 C. There are two sets of SbCWINV, with one set containing SbCWINV2/3/5/6, and the other set containing SbCWINV8/9/10, which are located in unassembled supercontig_67 and Chromosome 6, respectively. Both of the two sets of genes were observed to have originated from tandem duplications (Additional file 1: Table S1).

### Cloning and sequence analysis of INV gene family in sugarcane

Using the 19 *SbINVs* combined with sugarcane ESTs as reference for primer design, 11 cDNAs of the homologous INVs in sugarcane were cloned by RT-PCR. The 11 cDNAs are referred to as ShN/AINV1, 2–2, 3–2, 4–2, 5, 6–2, ShCWINV6, 7–3, 8–2, 10 and ShVINV1 according to both primers reference from SbINVs and sequences similar to SbINVs (Table 1). 3 sequences (ShCWINV6, ShCWINV8–2, ShVINV1) only harbour partial open reading frames (ORF), the remaining 8 were predicted to contain full ORFs (Table 2). Furthermore, to examine the gene structure of INVs in sugarcane, genomic PCRs were performed to clone sugarcane INVs. Twelve DNA fragments corresponding to 9 ShINVs were obtained, 8 of these sequences (in addition to ShCWINV3, ShCWINV8–1, ShCWINV9–1 and ShCWINV9–2) were determined to contain full coding regions (Table 2). Among the 9 ShINVs, ShCWINV7 and ShCWINV9 had 2 and 3 gene alleles, respectively. Overall, the 23 sequences including 11 cDNA and 12 genomics fragments corresponded to 14 INV genes including 6 N/AINVs, 7 CWINVs and 1 VINV. These DNA and cDNA sequences were submitted to Genbank: *ShN/AINV1* (KC145794), *ShN/AINV2–1* (KC145808), *ShN/AINV2–2* (KC145795), *ShN/AINV3–1* (KC145809), *ShN/AINV3–2* (KC145796), *ShN/AINV4–1* (KC145810), *ShN/AINV4–2* (KC145797), *ShN/AINV5* (KC145799), *ShN/AINV6–1* (KC145807), *ShN/AINV6–2* (KC145798), *ShCWINV1* (KC145815), *ShCWINV3* (KC145801), *ShCWINV6* (KC145800), *ShCWINV7–1* (KC145811), *ShCWINV7–2* (KC145812), *ShCWINV7–3* (KC145802), *ShCWINV8–1* (KC145814), *ShCWINV8–2* (KC145816), *ShCWINV9–1* (KC145803), *ShCWINV9–2* (KC145804), *ShCWINV9–3* (KC145813), *ShCWINV10* (KC145805), *ShVINV1* (KC145806) (Table 1).

**Table 1:** The information on PCR products of the invertase genes in sugarcane

Sorghum		Sugarcane DNA clone		Sugarcane cDNA clone		Protein coverage and similarity (%)
Gene name	Gene ID	Gene name	Gene ID	Gene name	Gene ID	
SbN/AINV1	Sobic.004G172700	N/A	N/A	ShN/AINV1	KC145794	N/A
SbN/AINV2	Sobic.004G255600	ShN/AINV2–1	KC145808	ShN/AINV2–2	KC145795	99%/98%
SbN/AINV3	Sobic.005G058800	ShN/AINV3–1	KC145809	ShN/AINV3–2	KC145796	100%/99%
SbN/AINV4	Sobic.004G024500	ShN/AINV4–1	KC145810	ShN/AINV4–2	KC145797	100%/98%
SbN/AINV5	Sobic.004G163800	N/A	N/A	ShN/AINV5	KC145799	N/A
SbN/AINV6	Sobic.003G153800	ShN/AINV6–1	KC145807	ShN/AINV6–2	KC145798	100%/99%
SbN/AINV7	Sobic.001G391600	N/A	N/A	N/A	N/A	N/A
SbCWINV1	Sobic.001G099700	ShCWINV1	KC145815	N/A	N/A	N/A
SbCWINV2	Sobic.K040900	N/A	N/A	N/A	N/A	N/A
SbCWINV3	Sobic.K041100	ShCWINV3	KC145801	N/A	N/A	N/A
SbCWINV4	Sobic.004G166700	N/A	N/A	N/A	N/A	N/A
SbCWINV5	Sobic.K041000	N/A	N/A	N/A	N/A	N/A
SbCWINV6	Sobic.K041200	N/A	N/A	ShCWINV6	KC145800	N/A
SbCWINV7	Sobic.003G440900	ShCWINV7–1	KC145811	ShCWINV7–3	KC145802	99%/92%
		ShCWINV7–2	KC145812			99%/93%
SbCWINV8	Sobic.006G255500	ShCWINV8–1	KC145814	ShCWINV8–2	KC145816	85%/98%
SbCWINV9	Sobic.006G255400	ShCWINV9–1	KC145803	N/A	N/A	N/A
		ShCWINV9–2	KC145804	N/A	N/A	N/A
		ShCWINV9–3	KC145813	N/A	N/A	N/A
SbCWINV10	Sobic.006G255600	N/A	N/A	ShCWINV10	KC145805	N/A
SbVINV1	Sobic.004G004800	N/A	N/A	ShVINV1	KC145806	N/A
SbVINV2	Sobic.006G160700	N/A	N/A	N/A	N/A	N/A

**Table 2:** Comparison of the characterisation of the invertases between sugarcane and sorghum

Sorghum						Sugarcane						Coverage/identity
Gene name	Protein size(aa)	MW (kDa)	pI	Subcellular localization		Gene name	Protein size(aa)	MW (kDa)	pI	Subcellular localization		
				SignalP/Chlor-oP/MitoProt	Plant-mPLoc					SignalP/Chlor-oP/MitoProt	Plant-mPLoc	
SbN/AINV1	559	63.16	6.3	N/44.6^c^/22.3^m^	Chloroplast	ShN/AINV1	559	63.13	6.1	N/44.6^c^/23.8^m^	Chloroplast	100%/98%
SbN/AINV2	572	64.17	6.5	N/44.8^c^/3.1^m^	Chloroplast	ShN/AINV2–1	567	63.58	6.0	N/44.3^c^/2.3^m^	Chloroplast Mitochondrion	99%/97%
						ShN/AINV2–2	575	64.58	6.5	N/45.6^c^/4.1^m^	Chloroplast Cytoplasm	100%/97%
SbN/AINV3	558	63.65	6.2	N/43.4^c^/6.2^m^	Chloroplast	ShN/AINV3–1	557	63.58	6.2	N/43.5^c^/22.1^m^	Chloroplast	100%/99%
						ShN/AINV3–2	557	63.58	6.2	N/43.5^c^/2.1^m^	Chloroplast	100%/99%
SbN/AINV4	627	70.51	7.9	N/43.0^c^/0.3^m^	Chloroplast	ShN/AINV4–1	563	64.06	6.7	N/43.1^c^/0.1^m^	Chloroplast Nucleus	100%/98%
						ShN/AINV4–2	563	63.90	6.7	N/43.1c/0.3 m	Chloroplast	100%/97%
SbN/AINV5	603	67.93	6.3	N/54.9^c^/89.7^m^	Chloroplast	ShN/AINV5	607	68.28	6.3	N/55.2^c^/97.9^m^	Chloroplast	100%/97%
SbN/AINV6	627	70.06	5.4	N/55.3^c^/98.6^m^	Chloroplast	ShN/AINV6–1	623	69.67	5.4	N/53.1^c^/99.6^m^	Chloroplast	99%/98%
						ShN/AINV6–2	629	70.23	5.4	N/54.5^c^/99.2^m^	Chloroplast	100%/98%
SbCWINV1	579	63.25	5.9	Y/46.2^c^/56.1^m^	Cell wall	ShCWINV1	572	62.79	6.2	Y/45.7^c^/37.2^m^	Cell wall	98%/94%
SbCWINV3	594	65.67	9.3	N/43.8^c^/3.8^m^	Cell wall	ShCWINV3^*^	453	50.21	9.4	N/44.1^c^/12.2^m^	Cell wall	100%/92%
SbCWINV6	599	66.90	9.5	N/44.5^c^/2.5^m^	Cell wall	ShCWINV6^*^	349	39.84	9.5	N/44.0^c^/53.5^m^	Cell wall	66%/90%
SbCWINV7	643	73.06	6.7	Y/50.9^c^/87.6^m^	Cell wall	ShCWINV7–1	588	66.40	6.4	Y/50.4^c^/72.9^m^	Cell wall	99%/90%
						ShCWINV7–2	598	67.84	6.2	Y/50.7^c^/69.2^m^	Cell wall	99%/90%
						ShCWINV7–3	593	67.13	6.3	Y/51.1^c^/85.3^m^	Cell wall	99%/90%
SbCWINV8	556	61.44	5.4	N/44.6^c^/49.4^m^	Cell wall	ShCWINV8–1^*^	501	55.60	5.4	N/44.1^c^/41.5^m^	Cell wall	88%/92%
						ShCWINV8–2^*^	448	49.46	5.0	N/46.7^c^/9.4^m^	Cell wall	76%/93%
SbCWINV9	590	64.62	5.3	Y/46.3^c^/9.7^m^	Cell wall	ShCWINV9–1^*^	211	23.08	5.3	N/44.3^c^/8.9^m^	Cell wall	40%/94%
						ShCWINV9–2^*^	503	54.76	4.9	N/44.6^c^/5.3^m^	Cell wall	89%/89%
						ShCWINV9–3	574	62.60	5.1	Y/48.1^c^/9.1^m^	Cell wall	100%/91%
SbCWINV10	625	68.30	5.2	Y/51.5^c^/25.3^m^	Cell wall	ShCWINV10	592	64.39	6.0	Y/50.1^c^/0.5^m^	Cell wall	99%/87%
SbVINV1	638	69.32	5.7	N/46.3^c^/8.3^m^	Vacuole	ShVINV1^*^	487	52.94	5.0	N/46.3^c^/5.0^m^	Vacuole	100%/94%

Note: ^m^ Probability (%) of targeting to mitochondrion, ^c^ Probability (%) of targeting to chloroplast; N--Non-secretory protein, Y-- Secretory protein, ^*^ represented truncated gene

These INV sequences were translated into amino acid sequences for computational analysis of protein characteristics (Table 2). Of these 23 ShINV sequences, 16 ShINV sequences containing complete ORFs (open reading frames) were predicted to have molecular weights ranging from 62.60 to 70.23 kDa (Table 2). Comparative analysis of the protein sequences of orthologous sorghum genes showed, neutral/alkaline INVs shared higher identities (ranging from 97 to 99%) than the acid INVs (ranging from 87 to 94%), indicating that faster divergence occurred in neutral/alkaline INVs than in acid INVs after the split of sugarcane and sorghum (Table 2). The molecular mass of the homologous INV proteins in sugarcane and sorghum were similar excluding those sequences without full ORFs (Table 2). Based on predictions of the subcellular localization by Plant-mPLoc, INVs were divided into three types: cell-wall, vacuolar, and cytoplasmic. For neutral/alkaline INVs, except ShN/AINV2–1 and ShN/AINV4–1, the other neutral/alkaline INVs were predicted to localize to the chloroplast. However, the localization probability predicted by ChloroP/MitoProt suggested that five neutral/alkaline INV proteins (SbN/AINV5, ShN/AINV5, SbN/AINV6, ShN/AINV6–1, ShN/AINV6–2) most likely localize to the mitochondria, since the probability of mitochondrial targeting was higher than chloroplast targeting (Table 2). In addition, using the online tool SignalP, four sets of orthologous genes of sugarcane and sorghum (CWINV1s, CWINV7s CWINV9s and CWINV10s) were predicted to possess the hydrophobic N-terminal signal peptide required for secretory proteins (Table 2).

Because *Saccharum* hybrids are highly allopolyploid with genetic backgrounds from *S.officinarum and S.spontaneum*, the sequences for gene alleles could derive from either of these two *Saccharum* species. Of the 23 sequences for 14 INV genes, 7 *ShINV*s (*ShN/AINV2*, *ShN/AINV3, ShN/AINV4*, *ShN/AINV6*, *ShCWINV7*, *ShCWINV8* and *ShCWINV9*) had 2–3 gene alleles. Of these 7 *ShINV*s, the alleles of *ShN/AINV2*, *ShN/AINV3*, *ShN/AINV4* and *ShN/AINV6* shared protein sequence similarities ranging from 98% to 99% (Table 3). The alleles of *ShCWINV7* shared protein sequence identities ranging from 92 to 93%, and *ShCWINV9–1*, *ShCWINV9–2* and *ShCWINV9–3* shared protein sequences ranging from 91 to 97%, while the protein sequence identity of the alleles *ShCWINV8–1* and *ShCWINV8–2* was 98%. These results indicated that the alleles of *ShN/AINV2*, *ShN/AINV3*, *ShN/AINV4*, *ShN/AINV6*, and *ShCWINV8* probably originated from *S. officinarum,* which contributed approximately 80% of genetic background, while the gene alleles of *ShCWINV7* and *ShCWINV9* could be derived from the two *Saccharum* species since the alleles within each genes presented sequence variation (Table 4).

**Table 3:** Amino acid sequence pairwise comparisons (% similarity) between neutral/ alkaline INV members in sugarcane

	ShN/AINV1	ShN/AINV2–1	ShN/AINV2–2	ShN/AINV3–1	ShN/AINV3–2	ShN/AINV4–1	ShN/AINV4–2	ShN/AINV5	ShN/AINV6–1	ShN/AINV6–2
ShN/AINV1										
ShN/AINV2–1	77									
ShN/AINV2–2	79	98								
ShN/AINV3–1	75	69	70							
ShN/AINV3–2	75	69	70	99						
ShN/AINV4–1	65	67	67	69	69					
ShN/AINV4–2	65	66	66	67	67	98				
ShN/AINV5	52	57	57	61	61	54	54			
ShN/AINV6–1	60	60	61	62	62	57	57	74		
ShN/AINV6–2	60	60	60	62	62	57	56	73	99

**Table 4:** Amino acid sequences pairwise comparisons (% similarity) between acid INV members in sugarcane

	ShCWINV1	ShCWINV3^*^	ShCWINV6^*^	ShCWINV7–1	ShCWINV7–2	ShCWINV7–3	ShCWINV8–1^*^	ShCWINV8–2^*^	ShCWINV9–1^*^	ShCWINV9–2^*^	ShCWINV9–3	ShCWINV10	ShVINV1^*^
ShCWINV1													
ShCWINV3^*^	59												
ShCWINV6^*^	61	80											
ShCWINV7–1	51	49	53										
ShCWINV7–2	51	49	53	93									
ShCWINV7–3	51	49	53	92	93								
ShCWINV8–1^*^	48	46	47	51	52	50							
ShCWINV8–2^*^	47	47	47	50	51	50	98						
ShCWINV9–1^*^	63	49	57	56	56	56	72	71					
ShCWINV9–2^*^	49	46	48	48	48	48	61	62	97				
ShCWINV9–3	49	49	49	50	50	50	64	64	93	91			
ShCWINV10	50	49	51	52	51	51	66	65	70	64	64		
ShVINV1^*^	42	40	47	38	37	37	40	41	50	40	41	40	

### Gene structural and phylogenetic analysis of the INV genes in sugarcane and sorghum

Phylogenetic analysis of the INV gene family from sugarcane and sorghum showed that INVs can be divided into two branches (acid INV branch and neutral/alkaline INV branch), and each of these branches can be further subdivided into α and β subgroups (Fig. 4). Acid INV branch contained *CWINV* and *VINV*, the group of *CWINV* could be further divided into two subgroups (α and β). In subgroup α, three *CWINV*s (*ShCWINV1*, *ShCWINV3* and *ShCWINV7*) from sugarcane had a very conserved gene structure with their orthologous genes in sorghum, and among them ShCWINV7 had two gene alleles whereas ShCWINV7–1 has 10 amino acids fewer than ShCWINV7–2 and one exon more than ShCWINV7–2 (Table 2, Fig. 4). In subgroup β, ShCWINV8 shared a similar gene structure with its orthologous genes in sorghum, although its homologous gene SbCWINV8 contains one more 9 bp exon and one more last exon, while the three gene alleles of ShCWINV9 were observed to have exon splits in the third corresponding exon of SbCWIN9 in sorghum as shown by the gene structure of ShCWINV9–3 with full CDS.

In neutral/alkaline INV branch, the genes from subgroups α and β contained 6 and 4 exons, respectively (Fig. 4). In the α subgroup, genes were observed to present variation in intron size, whereas *ShN/AINV6–1* had the same gene structure as its orthologous gene *SbN/AINV6* in sorghum. In the β subgroup, genes showed conserved exon size, consequently, the three *ShN/AINV*s (*ShN/AINV2–1*, *ShN/AINV3–1* and *ShN/AINV4–1*) shared same intron-exon structures with their sorghum orthologous genes. *N/AINVs* were more conserved than *AINVs* according to the comparative analysis of sorghum and sugarcane*,* which is consistent with the above comparative analysis for orthologous gene pairs between sorghum and sugarcane based on sequence similarity.

### Motif distribution in sugarcane and sorghum invertases

To compare the INV functional domain between sugarcane and sorghum, we employed the MEME web server combined with DNAMAN to identify the motifs of INVs from sugarcane and sorghum. There were 15 conserved motifs identified in the INVs (Additional file 4: Table S4), of these acid INV motifs, motif 12, motif 7 and motif 14 contained the catalytic residues NDPN, RDP and WECP/VD respectively [29]. In general, excluding the sugarcane INVs lacking full CDS, the ShINVs harbored motif sequences similar to the orthologous INVs in sorghum except for a slight variation in one pair of orthologous genes (ShCWINV10/SbCWINV10). In ShCWINV10/SbCWINV10, motif 10 was absent in ShCWINV10 but present in SbCWINV10 (Fig. 5, Additional file 5: Figure S2 and Additional file 6: Figure S3). DPN in the NDPN motif is encoded by the mini-exon and is susceptible to alternative splicing under cold stress in potato with transfructosylating capabilities [6, 43], but it was absent from ShCWINV3*, ShCWINV6*, SbCWINV7/ShCWINV7–1/ShCWINV7–2, ShCWINV8–1*/ShCWINV8–2*, ShCWINV9–1*/ShCWINV9–2*/ShCWINV9–3 and SbVINV1/ShVINV1* (Additional file 5: Figure S2).

Neutral/alkaline INVs from sugarcane and sorghum were conserved for the putative functional motifs of their orthologous genes. Similarly, among the paralogous genes, the neutral/alkaline INVs from both sugarcane and sorghum were generally conserved for the motif distributions except for three motifs (motifs 13, 14 and 15) in the N-terminus. 12 motifs, including motif 3 and motif 6 which contained catalytic residues (two Asps) [21, 23], were observed to be consistent in their sizes and distributions (Fig. 5b, Additional file 6: Figure S3). Of the three variant motifs (motifs 13, 14 and 15) at the N-terminus, motif 15 was distributed in the N-terminus of SbN/AINV1/ShN/AINV1, SbN/AINV2/ShN/AINV2–1,-2 and SbN/AINV3/ShN/AINV3–1,-2; motif 13 was specifically distributed in the N-terminus of SbN/AINV4/ShN/AINV4–1,-2; motif 14 was specifically distributed in the N-terminus of SbN/AINV6/ShN/AINV6–1,-2 (Fig. 5b). Based on these differences from motif comparison, N/AINVs could be classed into four subfunctional divergences. These motif variations may lead to different biological characteristics and functions.

### Phylogenetic analysis of the invertase gene family and other plant invertase homologs

To understand the evolutionary relationship among the INV genes in sugarcane, unrooted phylogenetic trees were constructed for acid INVs and neutral/alkaline INVs using protein sequences from eight plant species (Additional file 1: Table S1, Fig. 2 and Fig. 3). In addition, 8 phylogenetic trees were constructed using INVs from *Arabidopsis thaliana*, *Vitis vinifera*, *Carica papaya*, *Oryza sativa*, *Brachypodium distachyon*, *Zea mays*, *Sorghum bicolor* and *Saccharum,* respectively (Additional file 7: Figure S1). Comparison of the phylogenetic trees from these plants demonstrated that the phylogenetic relationships of INVs in these plants are consistent and conserved (Additional file 7: Figure S1). All INVs from these plants fall into the acid and neutral/alkaline INV classes. Acid INVs can be divided into vacuolar INVs and two cell-wall subgroups (α and β groups). Alkaline/neutral INVs can also be further subdivided into α and β subgroups (Additional file 7: Figure S1). The phylogenetic tree of acid INV genes from eight plant species formed four evident branches that were designated as group I to IV (Fig. 2). In the phylogenetic tree, vacuolar INVs (VINVs) were distributed in the distinct branch I, which consists of genes from both dicotyledons and monocotyledons, whereas cell-wall INVs (CWINVs) could be classed into three branches (referred to as II, III and IV respectively). Of the three branches for cell-wall INVs, branch II and IV were specifically comprised of genes from monocotyledons and dicotyledons respectively, while, branch III contained genes from both dicot and monocot. These results indicated that the genes in branch III were more ancient than the genes in the other two branches. Furthermore, monocotyledonous genes specific to branch IV contained two subgroups that were more distinct than the dicotyledonous genes specific to branch II, suggesting that the gene duplications that occurred in monocotyledons predated those in dicotyledons. Alternatively, this result may indicate that monocot plants have a higher gene evolutionary rate than dicotyledons. In sugarcane, ShVINV1 was distributed in group I, ShCWINV1, ShCWINV3 and ShCWINV6 were distributed in group III, and ShCWINV7–1/2/3, ShCWINV8–1/2, ShCWINV9–2/3 and ShCWINV10 were in group IV.

The phylogenetic tree of neutral/alkaline INV genes from eight plant species could be separated into two distinct groups, referred to as α group and β group (Fig. 3). In the α group, the genes were subdivided into two subgroups, α1 and α2, which contained N/AINVs from dicot and monocot. In the α2 subgroup, the genes could be separated into two branches, whereas the genes from dicotyledons were distributed in one of these branches, suggesting the gene duplications were more ancient in monocot than in dicot in this subgroup. The β group contained 8 subgroups (β1-β8), of these 8 subgroups, β1, β4 and β8 were only comprised of monocotyledon N/AINVs, the remaining 5 subgroups only contained dicotyldeon N/AINVs. It is interesting that each of the monocotyledon specific subgroup (β1, β4 and β8) contained genes from all of the examined monocotyldeon species, while the dicotyledon genes were more divergent and distributed in different subgroups. These results suggested that N/AINVs in the β group were more recent in monocotyledons than in dicotyledons. In sugarcane, ShN/AINV5 and ShN/AINV6–1/2 were distributed in α1 and α2, respectively. ShN/AINV4–1/2, ShN/AINV3–1/2 and ShN/AINV1 were distributed in β1, β2, and β3, respectively.

### Expression of INVs under PEG stress, cold stress and sugar treatments

To test how INVs respond to drought and low temperature stress, and to illustrate whether the expression of INVs is regulated by the hydrolysis products (glucose and fructose) or by its substrate (sucrose), we examined the transcription levels of the 13 INVs in 1-month-old sugarcane seedlings under PEG stress, cold stress and sugar (glucose, fructose and sucrose) treatments. This could also provide insight into the potential function divergence of the INV gene family members. Of the examined genes (Fig. 6), *ShCWINV3*, *ShCWINV7*, *ShCWINV9* and *ShN/AINV4* were found to be up-regulated (at least as two fold as controls) under PEG stress, cold stress and sugar treatments, *ShCWINV6* and *ShN/AINV6* were down-regulated under PEG stress, cold stress and sugar treatments. As for *ShVINV1*, its expression decreased 0.5 fold under cold treatment and increased nearly 2 folds under fructose treatment compared to the control, while it showed no change under other treatments (Fig. 6). *ShN/AINV3* showed no significant difference in expression in any of the treatments except for cold treatment. The other 5 INVs were observed to be remarkably dissimilar in terms of the relative expression levels under five treatments (Fig. 6). In PEG treatment, compared to the control, the expression of *ShCWINV3*, *ShCWINV7*, *ShCWINV8*, *ShCWINV9*, *ShN/AINV3*, *ShN/AINV4* and *ShN/AINV5* increased, with the most significant being *ShCWINV3* with an increase in excess of 3 fold. The expression of *ShCWINV6*, *ShCWINV10*, *ShN/AINV1*, *ShN/AINV2* and *ShN/AINV6* decreased, and with *ShN/AINV6* decreasing most significantly at 70% compared to the control (Fig. 6). In response to the cold treatment, acid INVs *ShCWINV3*, *ShCWINV7*, *ShCWINV8* and *ShCWINV9* were induced, while *ShCWINV6*, *ShCWINV10* and *ShVINV1* were repressed. In particularly, *ShCWINV3* had the largest up-regulation (greater than 5.5 fold) after cold treatment. All neutral/alkaline INVs were up-regulated (at least as two fold as the controls) in response to the cold treatment except *ShN/AINV6* that showed a slight degradation (Fig. 6). In treatments with three different sugars, the expressions of *ShCWINV3*, *ShCWINV7*, *ShCWINV9* and *ShN/AINV5* was up-regulated, in particular the expression of *ShCWINV7* and *ShCWINV9* increased about 4-fold, 6.5-fold and 4-fold expression under glucose, fructose and sucrose treatments respectively. In addition, *ShCWINV6* and *ShN/AINV6* were down-regulated under three sugar treatments, whereas no obvious change in expression was observed for *ShN/AINV1* and *ShN/AINV3* (Fig. 6).

Overall, transcripts of neutral/alkaline INVs were more abundant than transcripts of acid INVs. Among neutral/alkaline INVs, *ShN/AINV5* was the most abundant form and the expression level of *ShN/AINV2* under five treatments was the lowest. Among the acid INVs across the five treatments and control, *ShCWINV8* had significantly higher expression levels than the other genes, whereas *ShCWINV6* had the lowest expression levels. In addition, *ShCWINV1* displayed no expression in any of the five treatments and in the control (Fig. 6).

### Variation of enzymatic activity under drought, low temperature and sugar treatments in sugarcane

The activity of cell-wall INVs, soluble acid INVs and neutral/alkaline INVs was assayed in sugarcane seedlings under drought, low temperature and sugar treatments. Changes in the activity of all these INVs exhibited the same trend in response to the five different abiotic stresses (Fig. 7), in which the activity of all INVs decreased (Fig. 7). More specifically, compared to the control, cell-wall INV activity showed a gradual decrease (about 48%–66%) after five treatments. Soluble acid INV activity was reduced by 87% under PEG and fructose treatment, and up to 92% following sucrose treatment. In addition, neutral/alkaline INV activity revealed the smallest decrease among the three INV classes, with only a 53% decrease occurring upon sucrose treatment compared to the control. However, its activity decreased less than 50% decrease under other treatments (Fig. 7). Three kinds of invertase activity in different experimental conditions were significantly suppressed but comparatively speaking the suppression of soluble acid INV activity was more apparent. The neutral/alkaline INVs displayed high catalytic capacity in the control, with activity being 4.16 times higher than that of cell-wall INVs and 5.75 times higher than that of soluble acid INVs. However, the neutral/alkaline INVs with the highest catalytic capacity under five different treatments was more obvious, for example it showed 8.8 times higher activity than cell-wall INVs, and 19 times higher than soluble acid INV under PEG treatment. Under cold treatment, the hydrolytic activity of neutral/alkaline INVs was 6–7 times higher than cell-wall INVs and soluble acid INVs. Moreover, neutral/alkaline INVs had 8, 9.9 folds higher activity than cell-wall INV and soluble INVs under glucose treatment respectively and neutral/alkaline INVs had 27.6, 18.2 times higher activity than soluble acid INVs under sucrose and fructose treatment respectively (Fig. 7).

## Discussion

**Fig. 1:**
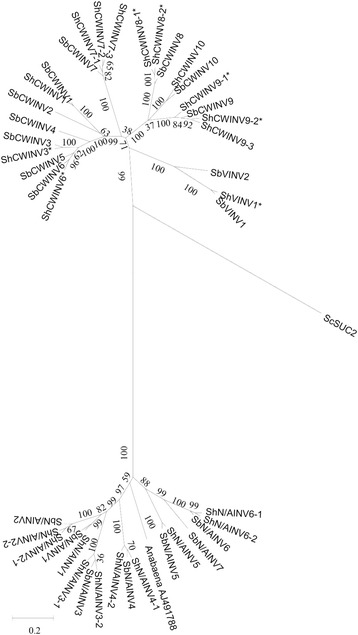
Phylogenetic tree of INVs in sugarcane and sorghum

INVs play a fundamental role in sucrose accumulation in plants, and have been well documented since half century ago in sugarcane [13, 17, 18]. Lack of a whole genome reference sequence for sugarcane made it difficult to determine the gene family members by PCR cloning using sorghum as reference, which is the closest relative of sugarcane with a reference sequence. In this study, eleven cDNAs and twelve DNAs corresponding to 14 sugarcane INVs were cloned based on the sorghum gene models (Additional file 1: Table S1, Table 1), thus providing the first study of the INV gene family in sugarcane. In sugarcane, PCR amplification of genomic and transcriptome based on the sorghum genome cannot capture highly divergent genes, making it impossible to determine the absolute number of INVs in sugarcane. Whole genome sequencing of sugarcane would be necessary for a comprehensive identification of a gene family in sugarcane (Fig. 1).

*Saccharum* hybrids are highly polyploid with a genetic background from *S.officinarum* and *S. spontaneum*. A typical gene in *Saccharum* can have up to 12 different alleles, each of which may be either from *S. officinarum* or *S. spontaneum* [57]. In this study, 5 of the 14 ShINVs had 2–3 gene alleles with variants for deduced amino acid sequences of each gene. Based on the sequence similarities among the gene alleles, it is difficult to determine the origin of the gene haplotype in *Saccharum* hybrids due to the close relationship between *S. officinarum* and *S. spontaneum.* Random PCR cloning from *Saccharum* hybrids for a gene functional study was not appropriate for the potential gene functional divergence between *S. officinarum* and *S. spontaneum*. Some gene alleles may have specific functions. For example, brown rust resistance gene (*Bru1*) in sugarcane was suggested to be single dose, which is the only resistant allele in Saccharum hybrid R570 [2, 12]. Future gene functional studies, should address the issue of identifying the gene alleles from *S. officinarum* contributing to the sugar characterization of *Saccharum* hybrids using the homologous genes of *S. officinarum* as a reference.

**Fig. 2:**
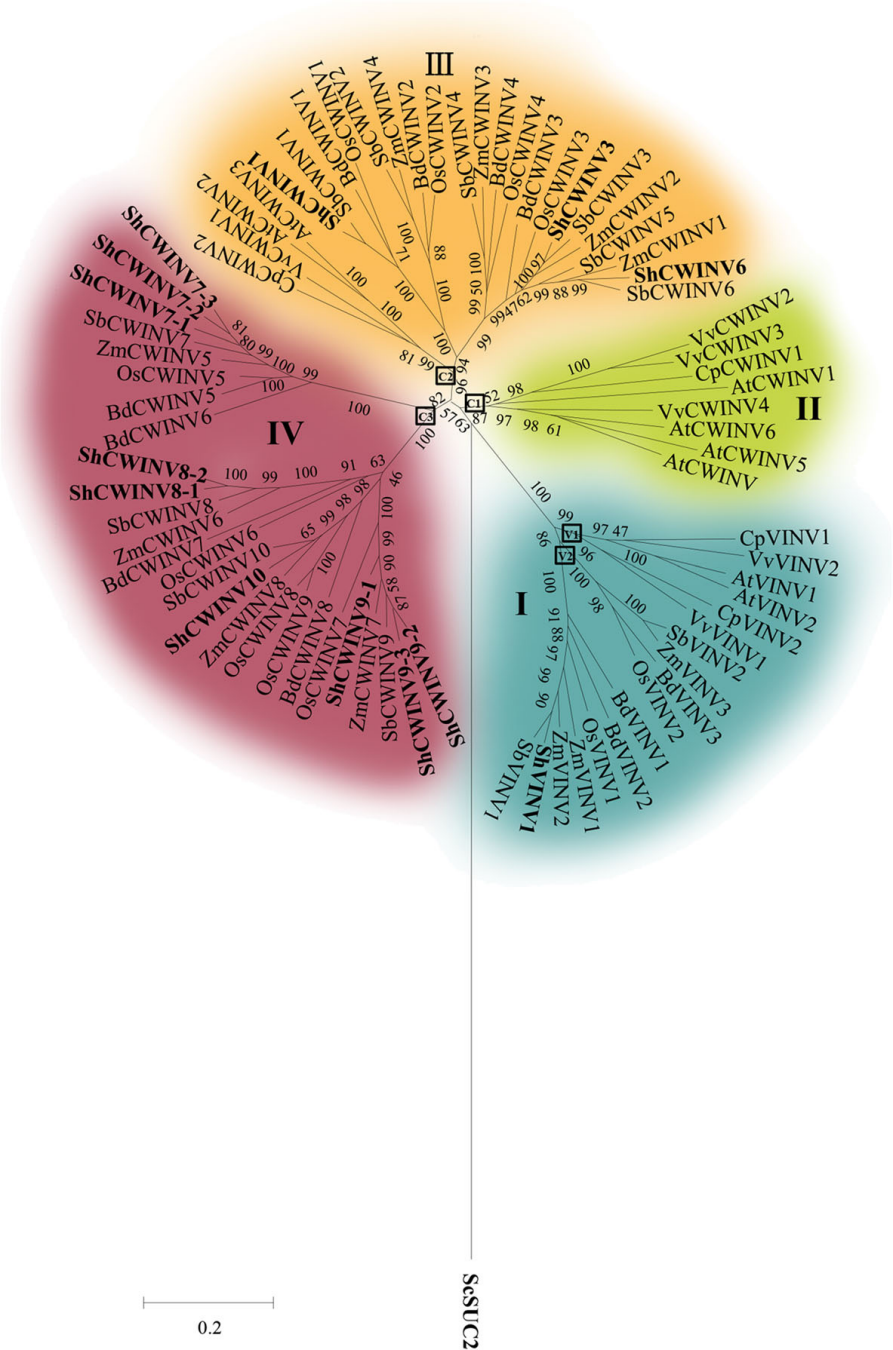
Phylogenetic tree of acid INVs from representative monocotyledons and dicotyledons. Analyses used the sequence of the central region of each protein, starting at the first conserved motif and ending at the last conserved motif. Yeast cell-wall invertase *Saccharomyces cerevisiae* SUC2 (NP_012104)

In this study, based on the phylogenetic analysis for acid INVs from eight plant species, acid INVs could be divided into four groups (I, II, III and IV, Fig. 2). All vacuolar INVs from both dicotyledons and monocotyledons were grouped in group I, which was distinctly separated from the cell-wall INV (Fig. 2), suggesting the origin of the vacuolar INVs from cell-wall INVs occurred before the last common ancestor (LCA) of dicotyledons and monocotyledons. This result broadened the previous deduction that the origin of vacuolar INVs from cell-wall INVs predated the LCA of rice and Arabidopsis [23]. The separation of the dicotyledon vacuolar INVs from the monocotyledon vacuolar INVs suggests that the LCA may contain a single vacuolar INV gene by two different pathways (gained or lost intron) to produce two different genes in the lineages to dicotyledons and monocotyledons, or the LCA may have possessed two vacuolar INV genes and the two precursors in LCA respectively underwent duplication events in each of the lineages to dicots and monocots. Both monocotyledons and dicotyledons had genes in group III, but monocots had higher numbers of the orthologous genes than dicots in group III, suggesting both a duplication exclusive to monocots and no potential gene functional redundancy in dicots. Furthermore, phylogenetic analysis suggested that the LCA of group III were more ancient than the LCAs of group II and group IV because group III was generated before monocot/dicot divergence. The comprehensive analysis also revealed the evolutionary history of acid INVs, which was sorted by age in duplicated descending order, group I (containing ShVINV1), group III (containing ShCWINV6, ShCWINV3, ShCWINV1), group IV (ShCWINV7, ShCWINV8, ShCWINV9). At least one gene duplication event was observed to have occurred in the four groups for acid INVs, suggesting the genes have potential functional redundancy. This speculation was consistent with the evidence from the comparison between the deduced amino acids of sorghum and sugarcane (Table 2).

**Fig. 3:**
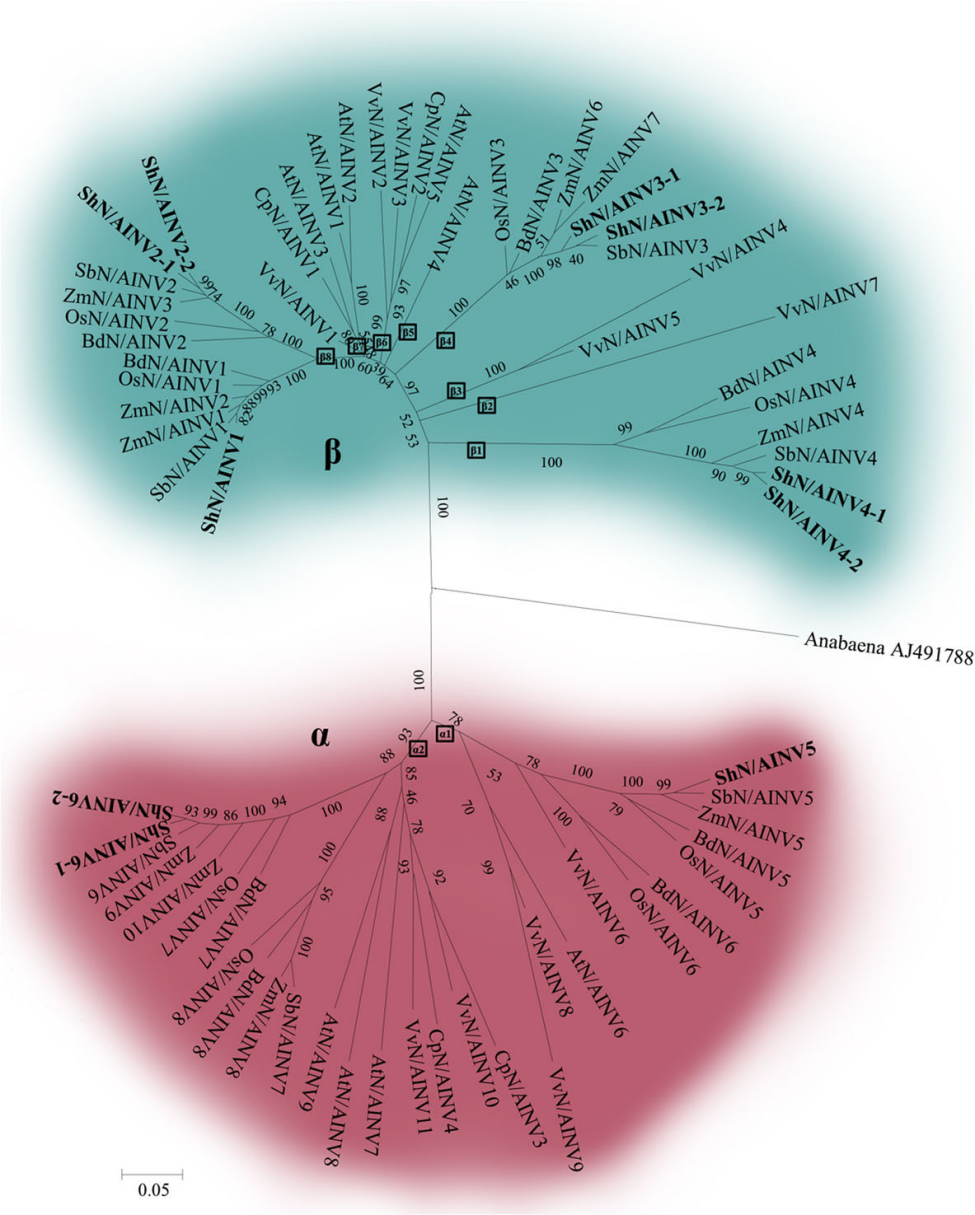
Phylogenetic tree of neutral/ alkaline INVs from representative monocot and dicot. Analyses used the sequence of the central region of each protein, starting at the first conserved motif and ending at the last conserved motif. The alkaline invertase of *Cyanobacterium Anabaena* (AJ491788) was used as an outlier

Analysis of the evolution of neutral/alkaline INVs for eight plants also revealed a similarly complex evolutionary relationship. In the phylogenetic tree of N/AINVs from eight plant species (Fig. 3), N/AINVs were distributed in two distinct groups (α and β groups). In the α group, the LCA for dicots and monocots contained two neutral/alkaline INV genes (boxes labeled α1 and α2). In the α1 lineage, a gene from grape (VvN/AINV6) was grouped together with monocot plant genes, while, in the α2 lineage, the genes from dicot and monocot were separated into two clear branches, and genes from monocot could also be subdivided into two branches. Therefore, the sugarcane genes (ShN/AINV6, and ShN/AINV7 (absent in sugarcane)) in the α2 lineage were suggested to be recent duplications after the split of dicot/monocot plants and were older than the sugarcane genes in the α1 lineage (ShN/AINV5). In the β group, LCAs of N/AINVs possessed eight genes (boxes labeled β1–β8), which occurred after the divergence of dicot and monocot plant species. Of the eight LCAs, β2 and β3 were the forerunners of grape N/AINVs, β5, β6 and β7 were the forerunners of dicot specific N/AINVs, β1, β4 and β8 were monocot specific forerunners of N/AINVs. It is interesting to note that all orthologous N/AINVs from monocot plants were grouped together, whereas, the dicotyledonous genes were grouped into β2 and β3 lineages which only contained grape genes, and β5 lineages which only contained one *Arabidopsis* gene. These results indicated that N/AINVs from the β group in dicot plants were more divergent than those in monocots. ShN/AINV1 and ShN/AINV2 in the β8 lineage were the most recently duplicated genes among their paralogous genes in the β group.

The orthologous gene pairs of N/AINVs between sorghum and sugarcane shared higher identities (97%–99%) than those of soluble acid INVs (87%–94%), demonstrating acid INVs had undergone a faster divergence than N/AINVs after the split of sorghum and sugarcane (Table 2). Sequence comparison of the paralogous gene in sugarcane also revealed that N/AINVs presented lower divergence than acid soluble INVs (Table 3). These results indicated that N/AINVs had undergone stronger functional constraint than acid soluble INVs. Sugarcane and sorghum had undergone 1 to 2 rounds gene duplications for LCAs of acid INVs after the splits of dicot/monocot, but only two sets of paralogous N/AINVs (SbN/AINV6/SbN/AINV6, ShN/AINV7 (absent in sugarcane) /SbN/AINV7) were recent duplications. Therefore, sorghum and sugarcane presented higher sequence variation for acid INVs than N/AINVs, which may be caused by functional redundancy of these acid INVs ancestors.

**Fig. 4:**
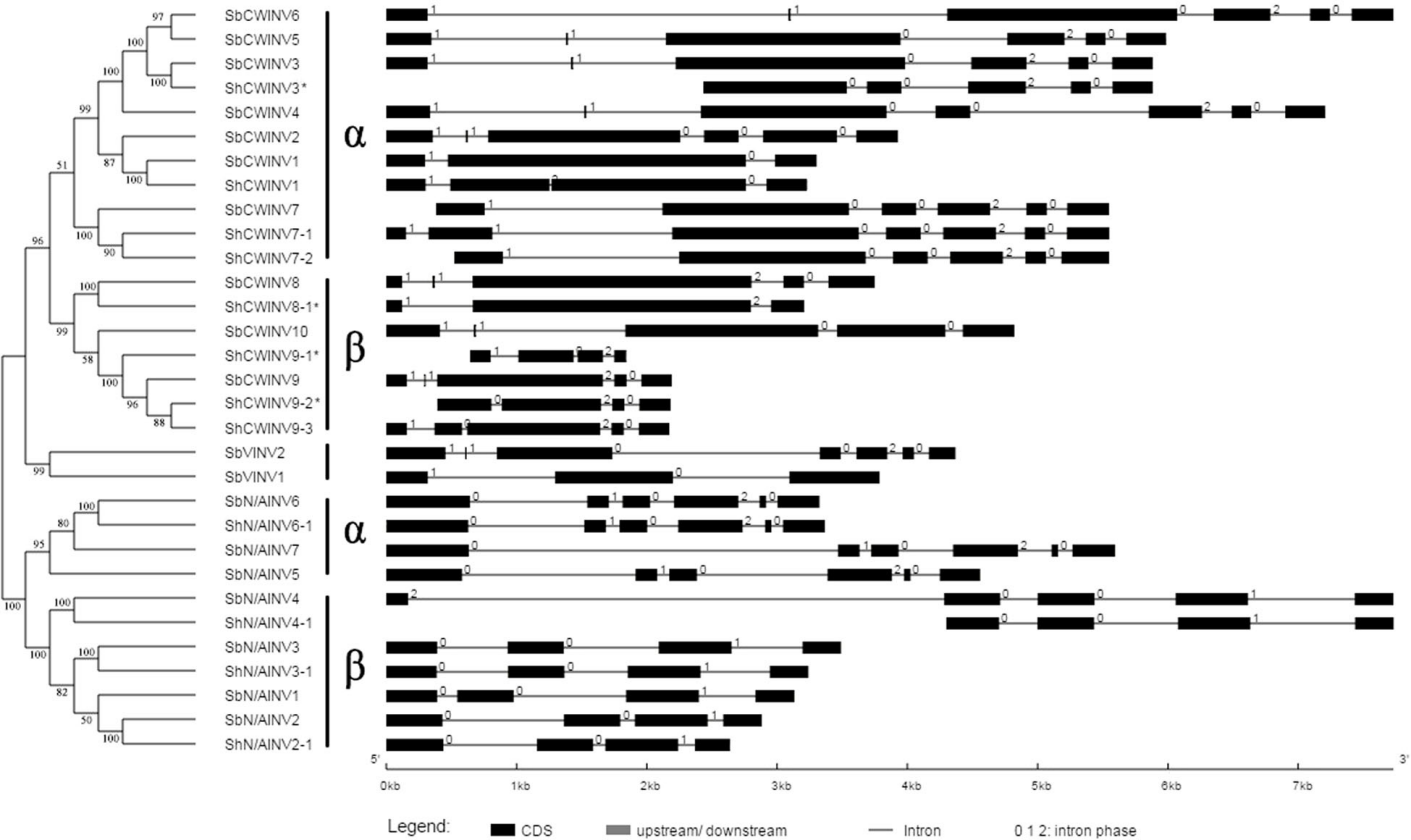
Comparison of phylogenetic tree and gene structure of invertases between sugarcane and sorghum. * represent the truncated genes

**Fig. 5:**
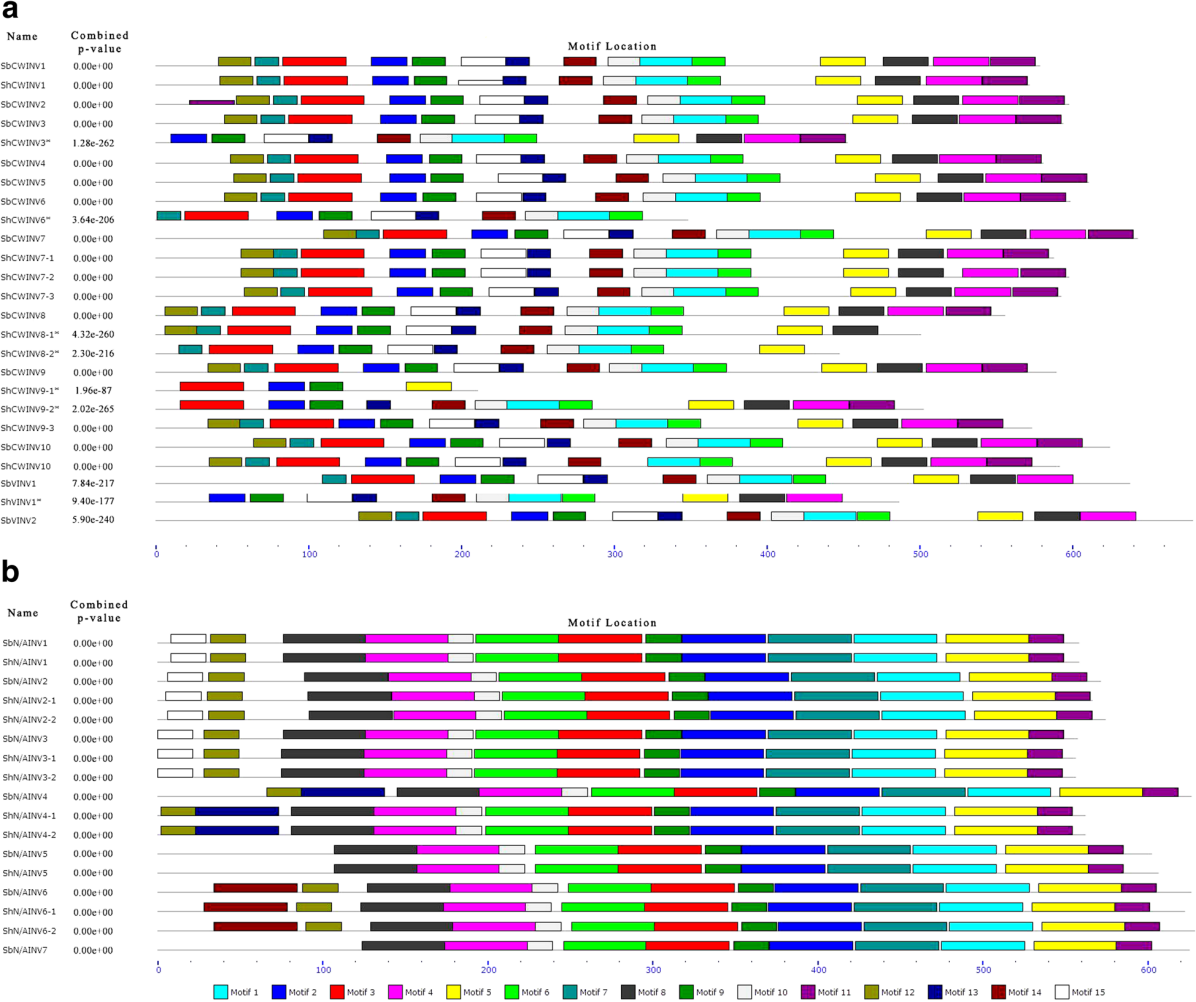
Motif distribution in invertases of sugarcane and sorghum. Motifs of acid invertases (**a**) and neutral/alkaline invertases (**b**) were investigated using the MEME web server. The different motifs are represented by different colours

To further understand the gene evolution of INVs, we analyzed the pattern of the exon–intron structure in sorghum and sugarcane showing that ShCWINV3, ShCWINV8–1, ShCWINV9–1 and ShCWINV9–2 underwent exons-loss (Fig. 4). Thus this evidence further confirmed that they were truncated genes, which was consistent with the analysis of amino acid sequences discussed above (Fig. 4, Additional file 5: Figure S2). Except these truncated genes, the exon–intron organizations of other genes were divergent among the CWINV gene families. The main motifs were kept in all the CWINV genes (Fig. 5), which suggested that the gene structure variation was caused by exon splitting or intron length variation but not pseudo-exonization as this would have resulted in motif deletion. The gene organization is highly conserved within the N/AINV gene family, and the evolution of neutral/alkaline INVs for sorghum and sugarcane was particularly clear in the sense that the locations of the exon–intron junctions in α group were distinct from the β group. All α group members had 6 exons and all β group members had 4 exons, with the exon–intron junctions being fully conserved. Therefore, the different intron-exon junctions and the different number of exons proved that the α and β groups of neutral/alkaline INV genes in sugarcane and sorghum derived from different ancestral genes with 4 and 6 exons, which is consistent with the findings reported in Fig. 3. Based on motif comparison, exonization may have occurred for the first exon of N/AINV gene because their first exons encode variant motifs. Overall, the N/AINVs have a more conserved gene structure than acid INVs, which also supported the above conclusion that acid INVs had undergone a faster divergence than N/AINVs after the split of sorghum and sugarcane.

**Fig. 6:**
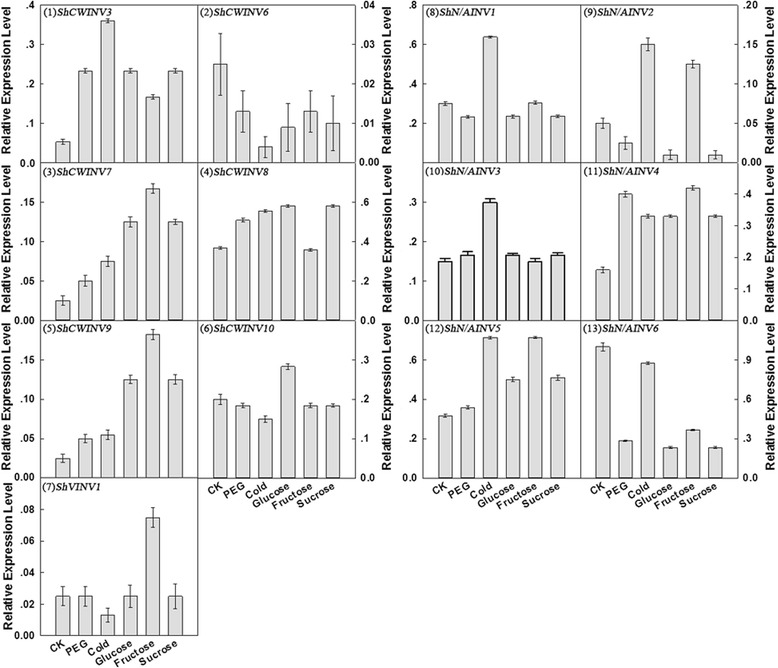
The expression patterns of INVs under drought, cold, exogenous sugars applications based on RT-q-PCR

The ability of sugarcane to accumulate sugar is impaired under drought stress and low temperature stress. We examined the expression patterns and activities of sucrose cleavage enzymes involved in sucrose metabolism under drought and low temperature stresses. These short-term physiological changes lead to sugar concentration changes, which may be significant enough to efficiently regulate gene expression. The expression of nearly all INV genes was affected by PEG and low-temperature treatment, except ShVINV1, whose expression was not altered in response to PEG. The observed up-regulation of expression of INV genes in sugarcane leaves in response to drought stress or cold treatment is likely due to more INVs being required to cleave sucrose into hexose sugars and subsequently provide cells with more energy to sustain increased respiration activity in addition to liberating more carbon and energy to synthesis different compounds [46], and enhance resistance to environmental stresses [53]. It is also possible that raised levels of INVs are required to cleave more Suc into Glc and Fru to greatly increase in the osmotic pressure of cells to cope with stresses, which indirectly provides evidence for INVs producing an osmoregulation substrate as in *Arabidopsis* [24]. Thus, it is possible that the expression of some INVs were up-regulated to cope with the need to cleave more sucrose under drought and cold stress, whereas the expression of other INVs could be down-regulated to maintain sucrose homeostasis. As shown in Fig. 6, four of the examined genes (ShCWINV3, ShCWINV7, ShCWINV8 and ShCWINV9) were up-regulated (especially ShCWINV3), and two genes (ShCWINV6 and ShCWINV10) were down-regulated under PEG and cold stress. Cell-wall INVs were thought to play a role in establishing metabolic sinks through irreversible cleavage of sucrose to glucose and fructose to metabolize sugars for both downstream metabolic functions and sugar modulation signaling [10, 38]. Whilst up-regulation of cell-wall INVs can easily be explained as a need to increase the ability for cleaving Suc into Glc and Fru, and enhance the osmotic pressure of cells, the down-regulated expression of ShCWINV6 might be due to a requirement for its distribution in specific subcellular compartments and interconnected with sugar modulation signal for blocking the downstream metabolism to adapt to the drought and cold stresses. The expression of ShN/AINV6 decreased under drought and cold stress, more specifically it decreased 3 fold under drought stress (Fig. 6). This result could be explained by the finding that the neutral/alkaline INVs may function as maintenance enzymes involved in sucrose degradation and maintenance of sucrose concentration [55]. In previous studies, soluble acid INVs were revealed to localize to the vacuole to control sucrose storage and sugar composition [48]. Under cold stress, all vacuolar INVs were observed to be up-regulated under cold stress in Populus [8] and the total INV mRNA levels were substantially upregulated in tulip (*Tulipa gesneriana* L. cv. Apeldoorn) bulbs [3]. However, in this study, ShVINV1 showed no changes in expression levels under drought stress, but was almost halved under cold stress. Thus, the down regulation of ShVINV1 and ShN/AINV6 under cold stress suggested that the difference of the molecular regulation mechanism between cold stress and PEG stress (Fig. 6). Sugar participates in numerous cellular processes. In addition to being a source of energy and form structural components during plant growth and development, it acts in signal transduction pathways to modulate the gene expression of sugar metabolism [46]. Glucose, sucrose as well as fructose have long been known as important signal molecules in the regulation of sugar accumulation [37]. To respond to changes in availability of sugars, cells can adjust the amount of invertases involved in sugar metabolism. Sugarcane grown on glucose, or fructose or sucrose as the sole carbon source display altered patterns of INV gene expression (Fig. 6). In response to sugar treatments, the INV genes in sugarcane presented different expression patterns consistent with the acid INV genes from maize in response to sucrose, glucose as well as other metabolizable sugars [56]. Previous studies on sucrose induction of INV expression have not addressed whether sucrose itself or its components (glucose and fructose) were the actual inducer [26]. However, recent studies on the regulation of INV by the nature of sugar signal molecules have revealed that sugar-specific pathways may be differentiated. For example, glucose, but not sucrose, induced the expression of cell wall INVs in Arabidopsis roots [55] and *C. rubrum* [38]. Moreover, the regulation of acid INV activity was repressed by hexose sugars, in particular by fructose [54]. However, in this study, both sucrose as a substrate of the INVs and also glucose induced the expression of 5 genes (ShN/AINV4, ShN/AINV5, ShCWINV3, ShCWINV7 and ShCWINV9). Exogenous sugars and sucrose repressed the expression of 2 genes (ShN/AINV6 and ShCWINV6) (Fig. 6).

**Fig. 7:**
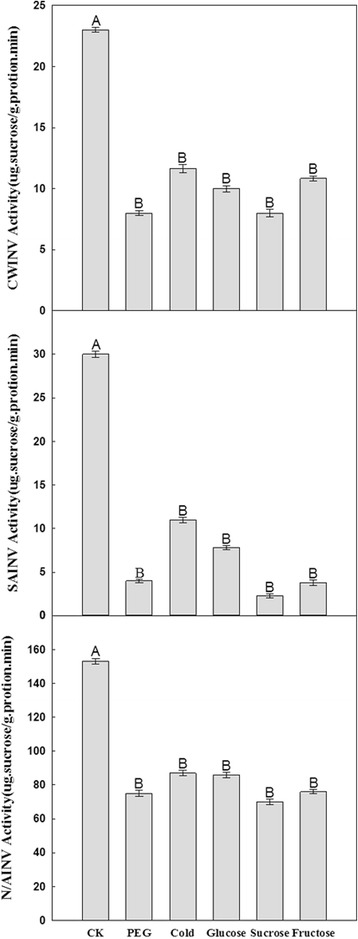
Effect of drought, cold and exogenous sugar applications on invertase activity in sugarcane seedlings. Note: The capital letter indicates utmost significantly different (*p* < 0.01)

These results suggested that sugars could directly regulate the expression of the majority of INV genes. In addition, nearly equally high levels of all invertase mRNAs, except ShCWINV10, were detected when the carbon source was either glucose or sucrose, which was lower than their expression with fructose, except for *ShCWINV3*, *ShCWINV8* and *ShN/AINV3* (Fig. 6). This finding indicated that the INV gene members have different sugar-specific response mechanisms; with the response of some INV genes to fructose being more sensitive than to sucrose or glucose. The general picture emerging from previous studies was that sugar signals decrease the transcription of genes for sugar synthesis and sucrose metabolism, and increase the transcription of genes involved in sugar storage and utilization [22, 28, 45, 55]. In agreement with this conclusion as well as our experimental results, we propose that in the presence of sufficient carbon sources regulating gene expression, ShN/AINV6 and ShCWINV6 may play a pivotal role in sugar synthesis and sucrose metabolism, and ShN/AINV4, ShN/AINV5, ShCWINV3, ShCWINV7 and ShCWINV9 may participate in sugar storage and utilization. Investigating gene expression and activities of INVs in sugar storage will be necessary to for further study these aspects. In general, the contributions of different INVs to the overall enzymatic activity of the INV family were different. In this study, activity of all INVs was significantly inhibited under five different abiotic stresses (Fig. 7). Figure 7 showed that neutral/alkaline INV had a much higher activity than soluble acid INV and cell-wall INV activity compared to the controls and other treatments. ShN/AINV6 with highest transcription levels in controls decreased sharply under the five abiotic stresses, leading to a possible decrease of neutral/alkaline INV activities (Fig. 6), suggesting that, it may provide the greatest contribution to the enzymatic activity of the whole neutral/alkaline INV family. Also in cell-wall INVs, ShCWINV8 with highest transcription levels in control may provide the largest contribution to the activity of the whole cell-wall INV family (Fig. 6). The expression of ShCWINV1 in control and under all stresses was not detected, and it cannot be excluded that its spatiotemporal expression pattern is very specific and was not captured in our experiments. We cloned only one member of the soluble acid INV genes, ShVINV1, and its expression levels were the same in all treatments except under cold and fructose stress (Fig. 6). Irrespective of the different experimental condition, its enzymatic activity was suppressed (Fig. 7). Thus it can be speculated that the decline in ShVINV1 enzymatic activity may be due to the expression of other genes, which are induced by abiotic stress and inhibit INV activity, or may be caused by structural modification(s) to the ShVINV1 of sugarcane. Together, both the gene expression patterns and enzyme activity changes under the biotic stress could help to further understand the interactive regulatory network of INV genes and sugar signaling pathways under drought, low temperature and exogenous sugars stresses. Noteworthy, cold may regulate gene expression, but cold itself also decreases the actual enzymatic activity in the plant. In our study, the activity of SAINVs under cold stress was higher than the other stresses in vitro. It is possible that the increase in enzyme occurs to maintain actual enzymatic activity under cold stress.

## Conclusion

This study is the first report for the 14 non-redundant members of the invertase gene family in sugarcane. We speculated that there were 6 neutral/alkaline invertases (ShN/AINVs) and 8 acid invertases (ShAINVs). We provided a comprehensive analysis of the gene allelic haplotypes, phylogenetic relationships, gene structure, functional domains, conserved motifs of proteins, gene expression patterns, and the variation of enzymatic activity under five abiotic stresses treatments for the INV gene family in sugarcane. Sequence comparison of the paralogous genes in sugarcane presented higher sequence variation for acid INVs than N/AINVs indicating that N/AINVs had undergone stronger functional constraint than acid soluble INVs. Furthermore, the N/AINVs have a more conserved gene structure than CWINVs, which also supports the idea that acid INVs have undergone a faster divergence than N/AINVs after the split of sorghum and sugarcane. Despite the high polyploidy level, the examined INV genes exhibited conserved gene structures and high similarity of amino acid sequences among the allelic haplotypes. Transcripts of neutral/alkaline INVs were more abundant than transcripts of acid INVs under drought stress, cold stress and sugar treatments. The expression of ShCWINV3, ShCWINV7, ShCWINV8 and ShCWINV9 were up-regulated to cope with the need to cleave more sucrose under drought and cold stress, whereas the expression of ShCWINV6 and ShCWINV10 could be down-regulated to maintain sucrose homeostasis. According to our experimental results, we also propose that sugars could directly regulate the expression of the majority of INV genes. In the presence of sufficient carbon sources regulating gene expression, ShCWINV9, ShCWINV3, ShCWINV7, ShN/AINV4 and ShN/AINV5 may critically participate in sugar storage and utilization, and ShN/AINV6 and ShCWINV6 may play a pivotal role in sugar synthesis and sucrose metabolism. In addition, all INVs’ activities were inhibited significantly under five different abiotic stresses. Based on the accrued data, we speculated that the contributions of neutral/alkaline INV to the overall enzymatic activity of the INV family were more than the sum of those of soluble acid INV and cell-wall INV activity. Further confirmatory experiments such as gene editing through the CRISPR-Cas9 system would be necessary to confirm this hypothesis. This study represents the first investigation of the INV gene family in sugarcane, providing the foundation to understand the physiological roles for each INV gene and unravel the molecular mechanism of sugar accumulation in sugarcane.

## Additional files

Additional file 1: Table S1. Basic information on invertase genes in 8 plant species. (XLS 49 kb)

Additional file 2: Table S2. PCR primer sequences and annealing temperatures of invertase genes in sugarcane. (PDF 13 kb)

Additional file 3: Table S3. Real time PCR primers of 13 invertase genes in sugarcane. (PDF 8 kb)

Additional file 4: Table S4. Motif distribution in the invertase family. (PDF 22 kb)

Additional file 5: Figure S2. Alignment of the conserved regions from known acid invertases of sorghum and sugarcane. (TIFF 2153 kb)

Additional file 6: Figure S3. Alignment of the conserved regions from neutral/alkaline invertases. (TIFF 1859 kb)

Additional file 7: Figure S1. Phylogenetic tree of INVs in monocotyledons and dicotyledons. (TIFF 8393 kb)

## Abbreviations

AINV: Acid invertase
BACs: Bacterial artificial chromosomes
CWINV: Cell-wall invertase
INV: Invertase
N/AINV: Neutral/alkaline invertase
SAINV: Soluble acid invertase
VINV: Vacuole invertases
