# Supplementary material for: Molecular cloning, structure, phylogeny and expression analysis of the invertase gene family in sugarcane
Source: BMC Plant Biol. 2017 Jun 23;17:109. doi: 10.1186/s12870-017-1052-0 (PMC5481874; doi:10.1186/s12870-017-1052-0)
Supplement: Supplementary file 2 — PCR primer sequences and annealing temperatures of invertase genes in sugarcane. (PDF 13 kb) [file 12870_2017_1052_MOESM2_ESM.pdf]

Table S2 PCR Primer Sequences and Annealing Temperature of Invertase Genes in Sugarcane

Sorghum		Forward primer and Reverse primer(5'-3')		Tm
Gene name	Gene ID			
SbN/AINV1	Sobic.004G172700	ATGGAGCTGGCTGTCGGCGGCGGGAT	TTAGTTTGTCCATGATGCGGACCGCTTCA	57
SbN/AINV2	Sobic.004G255600	ATGATGGAGGCGGCGGCGAT	TTACCCTGTCCAAGAGGCCGACCTCC	61
SbN/AINV3	Sobic.005G058800	TTTTCCCCCTGCTTTCAGTTTTGTT	CATGGTATACACTTTCTGTCCTATTT	58
SbN/AINV4	Sobic.004G024500	ATGGTACAATGTACTCAACCTCCTCCCCA	TCATGGGCACGAGTTGGAGCG	62
SbN/AINV5	Sobic.004G163800	AACAAGGCGCCAATGGGGATC	TGCTGCGAGAACTCACACAATGTATG	55
SbN/AINV6	Sobic.003G153800	ATGAGTGGTCAAACCCCGATGG	TCACCCAATATGTGACTTGGCTG	53
SbN/AINV7	Sobic.001G391600	ATGAGTGGTCAAACCCCGATGG	TCACCCAATATGTGACTTGGCTG	56
SbCWINV1	Sobic.001G099700	ATGGCGATCAGGACTTGGGCATCGGT	CTACTTCACGTCAGCGGTGATCTTAGCCGAC	59
SbCWINV2	Sobic.K040900	ATGAGTGGTCAAACCCCGATGG	TCACCCAATATGTGACTTGGCTG	58
SbCWINV3	Sobic.K041100	GATCATGTACACGGGCGTGAACCG	TCAAGCTCCATTCATGAGTGGCTTCTTC	59
SbCWINV4	Sobic.004G166700	ATGAGTGGTCAAACCCCGATGG	TCACCCAATATGTGACTTGGCTG	62
SbCWINV5	Sobic.K041000	ATGAGTGGTCAAACCCCGATGG	TCACCCAATATGTGACTTGGCTG	60
SbCWINV6	Sobic.K041200	ATGTACTACAAAGGGTGGTACCATTTC	CTAGATCTTGGCCCCGTTCATGA	59
SbCWINV7	Sobic.003G440900	ATGTCCATGGCCCTCAGGC	TAACTGTCTGTGCTTATCCCTTCTT	53
SbCWINV8	Sobic.006G255500	ATGAACGGGAAGCAGAGCCTACGCC	TTACAGTGTGCCCACACGGTAGTCTTCTTC	53
SbCWINV9	Sobic.006G255400	ATGGCAGCACTTCTCCTTGCTATACG	CTAATACATCTCGCTGTGGCACATGTTCA	59
SbCWINV10	Sobic.006G255600	CTCTCGTCCCGCATCGACCATG	TTAATAGGATTCACTTTCGTCTTTAGATTGAGAC	55
SbVINV1	Sobic.004G004800	ATGGTGCCGGACCAGTGGTA	TCAGATGTCCGTGACCATGTAGTCG	59
SbVINV2	Sobic.006G160700	ATGAGTGGTCAAACCCCGATGG	TCACCCAATATGTGACTTGGCTG	54
