# Supplementary material for: Molecular cloning, structure, phylogeny and expression analysis of the invertase gene family in sugarcane
Source: BMC Plant Biol. 2017 Jun 23;17:109. doi: 10.1186/s12870-017-1052-0 (PMC5481874; doi:10.1186/s12870-017-1052-0)
Supplement: Supplementary file 3 — Real time PCR primers of 13 invertase genes in sugarcane. (PDF 8 kb) [file 12870_2017_1052_MOESM3_ESM.pdf]

Table S3 Real time PCR primers for 13 invertase genes in sugarcane

Gene Name	Forward primer	Reverse primer
ShN/AINV6	GACCGTACCACTTGATGAGAACA	TCCACCACAGTCCAGAATCCA
ShN/AINV5	GGAACGGGACTTGGGTAAAGGA	AGACCACCATTGCCACCTACA
ShN/AINV4	TGCTGTGGCTGCTGGTGG	CCGCCTTGCCGTCGTAGT
ShN/AINV2	GCTCAACTACGACCAGGTGTTC	GCAGCAGCAGCGTCTTCA
ShN/AINV1	CGCCGCTCACTCGTCTTCT	CCCTCACAAACACTTGGTCGTAGT
ShN/AINV3	GTTGGTGAGATGCCTCTGAAGAT	GTCAGCAGCCACAGAAGAACT
ShCWINV10	GTGTCGTTGTCGTCTGCTCTTC	TCGGATCGTTCTGCCAGTTCT
ShCWINV9	GGAGCAGAAGCAGGACAAGGA	GGAGGCTGGTAGTGGTAGGC
ShCWINV7	GGCAGCAGGCAGCAGTAG	CCCACACGGTAGTCTTCTTCAAC
ShCWINV8	GGCTGAACTGGACGGCTACA	TGGTGGAGGCATTGGAAGAGT
ShCWINV3	GCCACCTGCGGTACGACTAC	AATCCCAGCCCAGCCCTTG
ShCWINV6	AGAGGCAAGGCGGTCACT	GCTAGGCGAGAAACGGTAACG
ShVINV1	GGCTGCCATCCTCATCATTATT	GGCGGATTGTGTGTGTAGTT
