# Supplementary material for: Molecular cloning, structure, phylogeny and expression analysis of the invertase gene family in sugarcane
Source: BMC Plant Biol. 2017 Jun 23;17:109. doi: 10.1186/s12870-017-1052-0 (PMC5481874; doi:10.1186/s12870-017-1052-0)
Supplement: Supplementary file 4 — Motif distribution in the invertase family. (PDF 22 kb) [file 12870_2017_1052_MOESM4_ESM.pdf]

Table S4 Motif distribution in invertase family

Invertase Family	Motif number	Length (aa)	Consensus sequence	E value
Acid invertase	1	34	RRILWGWANESDSEADDVAKGWAGIQAIPRTIWL	9.3e-537
	2	23	NYQVQNIAFPKNPSDPLLREWVK	4.1e-437
	3	41	WGNIVWGHSVSTDLINWIALEPAIEPTIPFDINGCWSGSAT	7.7e-384
	4	36	TISLRTLIDHSIVESFGQGGRTCIMSRVYPEHAITD	8.3e-433
	5	29	HGGVGPFGLWVMASGNMREHTAVFFRVFK	3.2e-410
	6	21	DPDGKQLLQWPVEEIETLRGK	7.3e-307
	7	15	YYKGMYHQFYQYNPH	5.8e-316
	8	29	LMCTDPTKSSTKPNLYKPTYGGFVDVDIE	2.0e-287
	9	21	PIIPPPQGINANQFRDPTTAW	1.4e-293
	10	21	WLRYDYGNFYASKTFFDPAKR	6.3e-267
	11	29	AHMYVFNNGTTDVKVSKLEAWEMKKAQMN	6.8e-255
	12	21	SPHYRTAYHFQPPKNWINDPN	2.9e-205
	13	15	SNATGMWECPDFFPV	1.4e-236
	14	21	TRCDYYMIGTYDDKTDTFVPD	3.9e-180
	15	29	SAEVDGYGAAYVYRSRDFKHWERVRHPLH	4.4e-112
Neutral/alkaline invertase	1	50	EMPLKICYPAMENHEWQIVTGCDPKNTRWSYHNGGSWPVLLWQLTAACIK	2.5e-736
	2	50	SYHMRSYYWLDFQQLNDIYRYKTEEYSHTAVNKFNVYPEQIPDWLFDFMP	2.4e-699
	3	50	RLILNLCLSEGFDTFPTLLCTDGCCMIDRRMGIYGYPIEIQALFYMALRC	4.9e-674
	4	50	NYDQVFVRDFVPSALAFLMNGETEIVKNFLLHTLQLQSWEKMVDCFKLGQ	3.3e-639
	5	50	ARRAIEVAEQRLAKDKWPEYYDGKLGRYVGKQARKFQTWSIAGYLVAKMM	2.7e-607
	6	50	ETLVADFGESAIGRVAPVDSGFWWIILLRAYTKWTGDNSLAETPDCQKGM	6.9e-607

7	50	GGYFIGNVQPAHMDFRWFCLGNCWAIVSSLATPEQAEAIMDLIEEKWDDL	7.7e-591
8	50	HTPASTHFNSFEPHPIVNEAWEALRRSVVYFCGQPVGTVAAVDHSCEEVL	2.6e-346
9	21	MLKPQNEGKDFIEHINTRLHA	4.0e-122
10	15	GAMPASFKVHHNPKD	4.6e-122
11	21	LEDPSHLMMISCEEDKHVKPP	1.3e-104
12	21	QRINVERQRSFDDRSWSELSH	7.0e-88
13	50	DENHDSLPKPEKRTRMHHIERHRSCVVTLSDMELNGLQPRRLLQTIEKSP	5.2e-61
14	50	KTAAGTATRLFPRLPHPHHPPYGRRLPFLVSAASQSQASQTGPATPVPVP	4.3e-58
15	21	MRRVSSHVSLADPDDFNLTRL	9.4e-42
