# Supplementary figures and images for: Molecular cloning, structure, phylogeny and expression analysis of the invertase gene family in sugarcane
Source: BMC Plant Biol. 2017 Jun 23;17:109. doi: 10.1186/s12870-017-1052-0 (PMC5481874; doi:10.1186/s12870-017-1052-0)

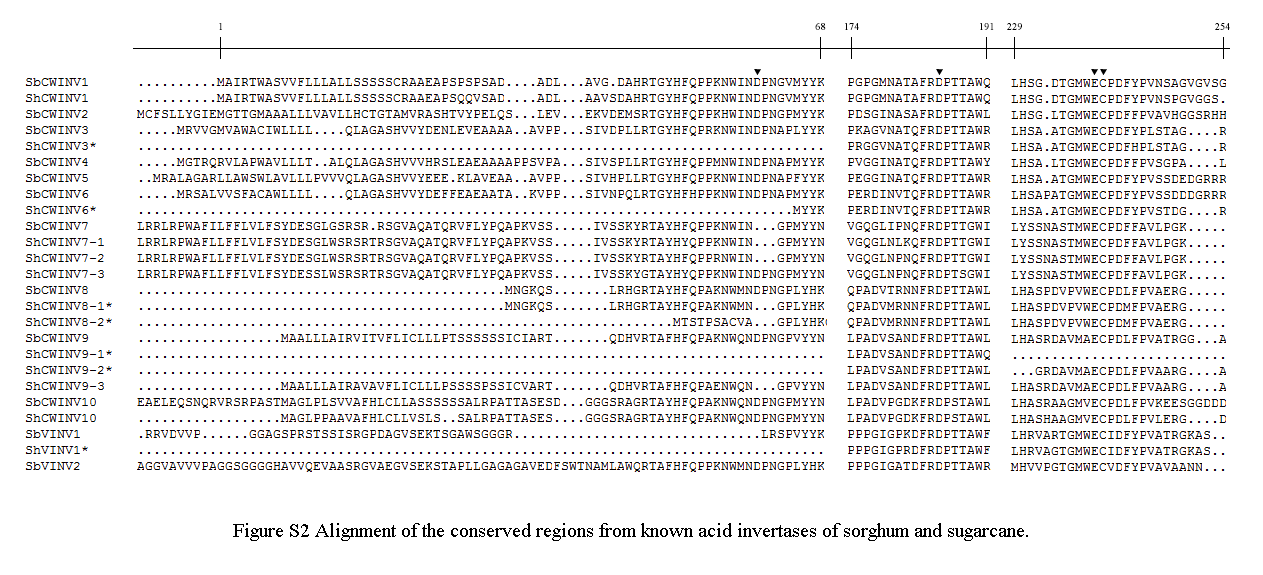

Supplement: Supplementary file 5 — Alignment of the conserved regions from known acid invertases of sorghum and sugarcane. (TIFF 2153 kb) [file 12870_2017_1052_MOESM5_ESM.tif]

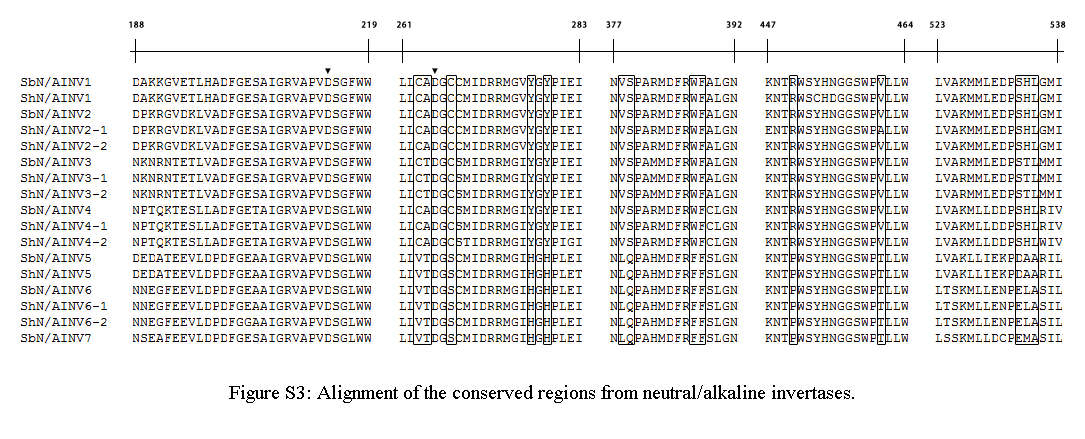

Supplement: Supplementary file 6 — Alignment of the conserved regions from neutral/alkaline invertases. (TIFF 1859 kb) [file 12870_2017_1052_MOESM6_ESM.tif]

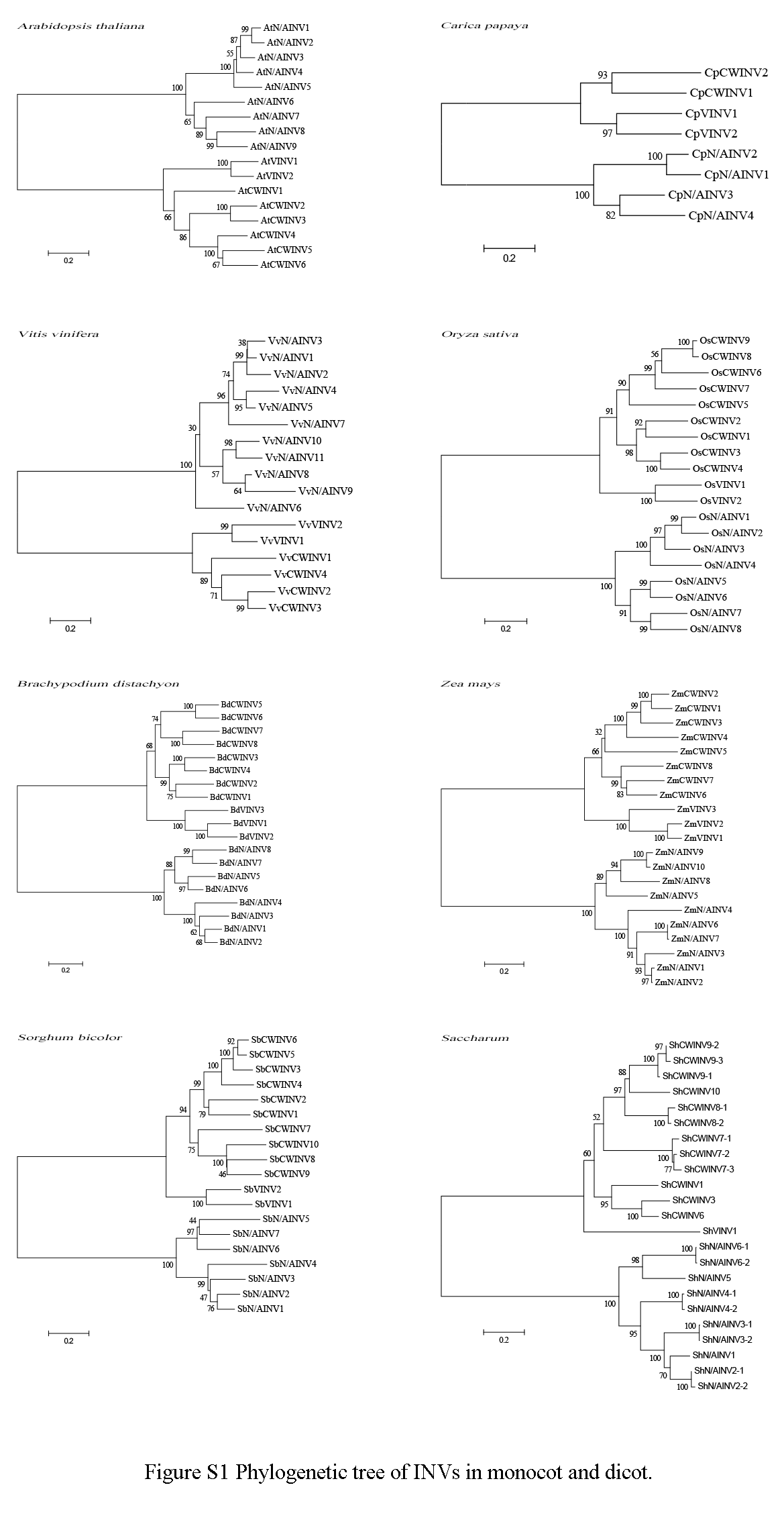

Supplement: Supplementary file 7 — Phylogenetic tree of INVs in monocotyledons and dicotyledons. (TIFF 8393 kb) [file 12870_2017_1052_MOESM7_ESM.tif]
